# Optical and Structural Property Tuning in Physical Vapor Deposited Bismuth Halides Cs_3_Bi_2_(I_1–*x*_Br_*x*_)_9_ (0 ≤ *x* ≤ 1)

**DOI:** 10.1021/acs.inorgchem.1c01545

**Published:** 2021-09-02

**Authors:** Sara Bonomi, Pietro Galinetto, Maddalena Patrini, Lidia Romani, Lorenzo Malavasi

**Affiliations:** † Department of Chemistry and INSTM, University of Pavia, Via Taramelli 16, Pavia 27100, Italy; ‡ Department of Physics, University of Pavia, Via Bassi 6, Pavia 27100, Italy

## Abstract

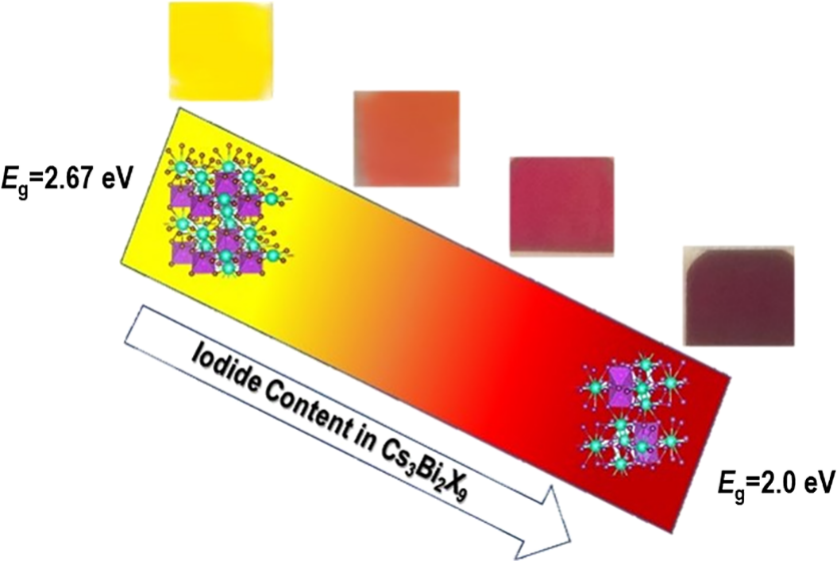

Crystalline films of lead-free all-inorganic Cs_3_Bi_2_X_9_ (X = Br, I) perovskites have been deposited by radio frequency (RF)-magnetron sputtering providing high-quality, single-phase films as confirmed by structural, morphological, and optical property characterization. Progressive tuning of crystal structure characteristics and optical absorbance has been achieved in mixed Br/I phases Cs_3_Bi_2_(I_1–*x*_Br_*x*_)_9_ (0 ≤ *x* ≤ 1), highlighting a shift of the band gap from about 2.0 eV for Cs_3_Bi_2_I_9_ to 2.64 eV for Cs_3_Bi_2_Br_9_. X-ray diffraction and Raman scattering allowed defining the range of alloyed compositions where single-phase compositions are found. Finally, preliminary photocatalytic activity tests on the degradation of methylene blue provided solid data indicating the future possible exploitation of Bi-based perovskite derivative materials as active photocatalysts.

## Abstract 2

High-quality films of the Cs_3_Bi_2_(I_1−*x*_Br_*x*_)_9_ (0 ≤ *x* ≤ 1) system have been grown by the vapor phase method for the first time. Crystal structure investigation revealed a phase change around *x* = 0.4, as demonstrated by X-ray diffraction and Raman spectroscopy. Halide alloying provided a continuous modulation of the band gap from 2.0 eV for Cs_3_Bi_2_I_9_ to about 2.64 eV for Cs_3_Bi_2_Br_9_.

## Introduction

Bismuth-based perovskite derivatives of general formula Cs_3_Bi_2_X_9_ (X = Br, I) are attracting huge interest from several diverse communities due to their technological potential spanning from photocatalysis to photodetectors, organic synthesis, and photovoltaics.^1−11^

Triggered by the issue of overcoming the concerns related to Pb-toxicity in metal halide perovskites (MHPs) for photovoltaics, the field of lead-free perovskites and perovskite derivatives has significantly extended toward novel applications by exploiting the photoactivity of lead-free materials which possess, in addition to relevant catalytic performance, water stability which is still a key problem of lead-based systems. Among Bi-containing perovskites, Cs_3_Bi_2_Br_9_ and Cs_3_Bi_2_I_9_ have been the object of several recent publications highlighting, in particular, their potential use in organic synthesis and photodetection.^5,6,8,11^ Cs_3_Bi_2_Br_9_ has a trigonal crystal structure (space group. S.G., *P*3̅*m*) characterized by a layered structure of alternating corner-sharing octahedra, and shows strong optical absorbance at around 400–500 nm depending on the material state, *i.e*. powder, single-crystals, or thin films.^12−16^ Its large excitonic binding energy prevents this material from being suitable for photovoltaics, nevertheless it has offered good performance as an active layer in photodetectors and, even more interestingly, in photocatalytic applications.^6,11,12^ Cs_3_Bi_2_Br_9_ was found to be effective in the direct selective photocatalyzed oxidation of hydrocarbons with high conversion rates and excellent selectivity, and in the ring-opening reaction of epoxides.^11^ In both cases, Cs_3_Bi_2_Br_9_ exhibited good stability and recyclability while its lead-based counterpart performed significantly worse. The Cs_3_Bi_2_I_9_ perovskite, crystallizing in a hexagonal symmetry (S.G., *P*6_3_/*mmc*), on the other hand, has been deeply investigated for its possible use as an active layer in perovskite solar cells (PSCs) since 2015, due to its high absorption coefficient and a band gap of about 2.10 eV.^1^ Strong effort has been put in place for optimizing the film morphology for PSCs due to the known dissolution problems of inorganic precursors when applying solution-based methods.^1^ The potential applications of Cs_3_Bi_2_I_9_ have been further extended in the last few years to photodetection and photocatalysis, in analogy with the bromide-containing phase.^5,6,9^ As an example, perovskite single-crystal thin films of Cs_3_Bi_2_I_9_ have been grown by a space-limited solvent evaporation crystallization method providing highly efficient photodetectors with impressive stability without any encapsulation for 1000 h in humid air (50% relative humidity (RH)).^17^ The same compound has been used in photocatalysis for hydrogen evolution and organic pollutant degradation.^18,19^

Mixed Cs_3_Bi_2_I_9–*x*_Br_*x*_ have also been the object of some investigation for both photovoltaics and photodetection applications.^2,5^ An extensive and deep study of the I/Br solid solution is reported in the work of Yu et al., where thin films of both end-members and intermediate mixed compositions have been prepared by spin coating and used in PSCs.^2^ While showing relatively low power conversion efficiencies (PCEs), with Cs_3_Bi_2_I_6_Br_3_ delivering 1.15% as the best-performing compound, the data confirm the significant stability of Bi-based phases.^2^ Liu et al., studied the photodetection performances of Cs_3_Bi_2_Br_9–*x*_I_*x*_ films by varying *x*, and achieved the best results for the Cs_3_Bi_2_I_6_Br_3_ composition with an excellent photosensitivity of 4.1 × 10^4^ at zero bias, as well as with the responsivity and detectivity reaching 15 mA W^–1^ and 4.6 × 10^11^ Jones.^5^ Moreover, in this case, excellent stability in the ambient environment, maintaining over 96% of the initial value after 100 days, was observed.^5^

All pieces of evidence reported so far refer to works carried out in the last couple of years suggesting that Bi-based layered perovskites show promise for future exploration and possible applications in several technologically relevant fields, as also recently demonstrated by the interest in tunable Cs_3_Bi_2_(Cl_1–*x*_I_*x*_)_9_ halide perovskites.^20^ In this respect, the possibility of film deposition scale-up is an urgent issue. Recently, physical vapor deposition methods have been triggering significant interest; they seem to be a valuable path to take the required step from the laboratory scale to the industrial scale in all of the fields where good quality thin films are required, in particular when considering all-inorganic perovskite materials where solution processing is more complicated with respect to hybrid organic–inorganic phases.^21^

Based on the above considerations and the strong appeal of Bi-based layered perovskites, in this work we carried out the vapor phase deposition, by means of radio frequency (RF)-magnetron sputtering, of the Cs_3_Bi_2_(I_1–*x*_Br_*x*_)_9_ system (0 ≤ *x* ≤ 1). To date, no vapor phase approaches have been used to prepare Cs_3_Bi_2_(I_1–*x*_Br_*x*_)_9_ films. In addition, RF-magnetron sputtering has scarcely been used for perovskite films notwithstanding its huge potential for scale-up, providing stoichiometry control, good morphology, and high crystallinity and quality of deposited films, as we demonstrated recently.^22,23^ Together with the structural and optical property characterization of the deposited films we report a significant photocatalytic activity in organic pollutant degradation combined with excellent water stability of the prepared materials. In the following, we start discussing the two end-members of the solid solution, namely Cs_3_Bi_2_Br_9_ and Cs_3_Bi_2_I_9_, and then moving to the investigation of the mixed compositions.

## Results and Discussion

### Cs_3_Bi_2_I_9_ and Cs_3_Bi_2_Br_9_ Perovskite Films

RF-magnetron sputtering has been adopted to deposit high-quality films of Bi-based layered perovskites starting with the preparation of Cs_3_Bi_2_I_9_ and Cs_3_Bi_2_Br_9_.

Cs_3_Bi_2_I_9_ films, with an average thickness of about 500–1000 nm (as determined by profilometry, see the Experimental Section), have been deposited on glass substrates starting from a target composed of stoichiometric amounts of CsI and BiI_3_ (see the Supporting Information, SI, for more details on the synthesis conditions) without substrate heating. Post-deposition thermal treatment has been carried out at 200 °C for 2 h under mild vacuum.

**Figure 1:**
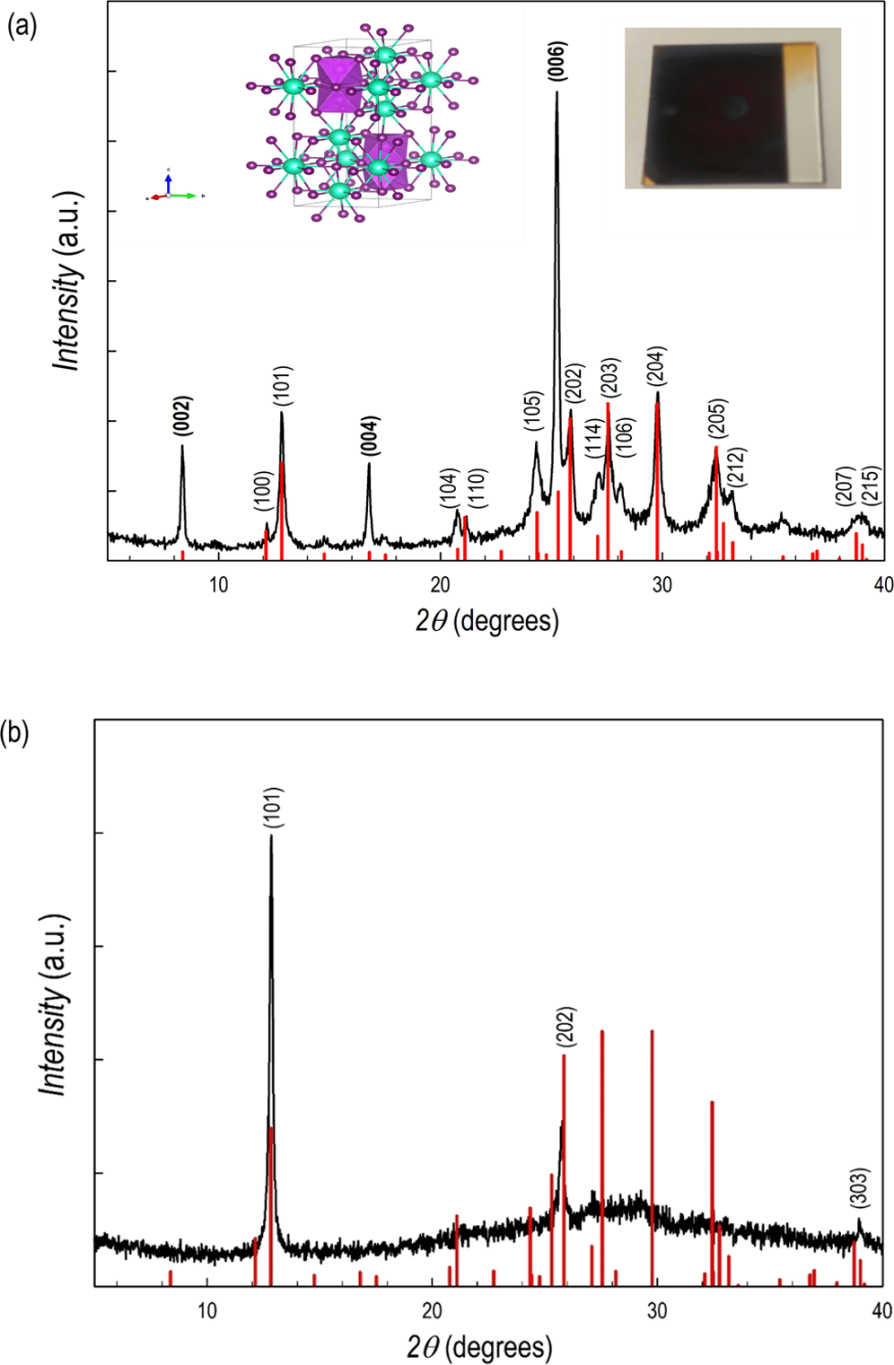
(a) XRD pattern of a Cs_3_Bi_2_I_9_ film superimposed to the calculated pattern of the hexagonal structure (vertical red lines), inset: right, photo of a Cs_3_Bi_2_I_9_ film deposited on a glass substrate; left: sketch of the crystal structure of Cs_3_Bi_2_I_9_; and (b) XRD pattern of a (*h*0*k*) fully oriented film of Cs_3_Bi_2_I_9_.

Characterization has been initially carried out by X-ray diffraction (XRD), revealing the crystal structure. Figure 1a displays the film pattern superimposed to the calculated diffraction for hexagonal (*P*6_3_/*mmc*—JCPDS card 01-070-0666) Cs_3_Bi_2_I_9_. There is a perfect match between experimental and calculated data confirming the deposition of single-phase films with good crystallinity as also evidenced by the narrow diffraction peaks. The lattice parameters are *a* = *b* = 8.4081(5) Å and *c* = 21.1520(8) Å. The inset of Figure 1a shows a picture of the film deposited by sputtering, which looks dark-red and highly reflective. A sketch of the hexagonal crystal structure is also reported in the inset of Figure 1a. In addition, a slight preferential orientation along the (00*l*) direction (Miller indexes in bold in Figure 1a) is observed by comparing the expected and experimental intensities. In addition, nearly (*h*0*l*) fully oriented films can be obtained by slightly changing the deposition conditions and, in general, such an effect is observed for films with a thickness of about 500–600 nm. A typical pattern of such an oriented film of Cs_3_Bi_2_I_9_ is shown in Figure 1b.

**Figure 2:**
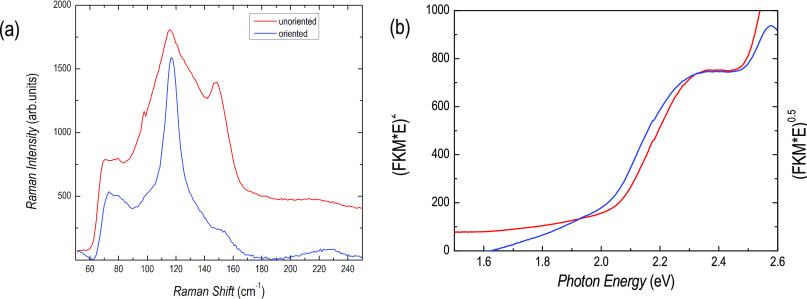
(a) Raman spectra of the unoriented (red line) and oriented (blue line) Cs_3_Bi_2_I_9_ films and (b) optical absorption edge of an oriented Cs_3_Bi_2_I_9_ film (direct gap red curve and indirect gap blue curve extrapolations).

Further insight into the film crystal quality has been obtained by Raman spectroscopy. Figure 2a shows the spectra of the films reported in Figure 1a,b. The spectrum of the unoriented film shows broadened Raman features with respect to reference Raman spectra of a single-crystalline sample of Cs_3_Bi_2_I_9_.^24^ Nevertheless, one can appreciate different peaks: according to ref (23), bridge Bi–I asymmetric stretching at around 90 cm^–1^, terminal Bi–I asymmetric stretching at around 120 cm^–1^, and the terminal Bi–I symmetric stretching at about 150 cm^–1^, are clearly visible, while other vibrational modes are less defined. On the other hand, for the oriented film, an intrinsic mode selection leads to a Raman spectrum dominated by the signal of asymmetric stretching at around 120 cm^–1^.

Finally, the optical response of Cs_3_Bi_2_I_9_ sputtered films have been determined by absorption and diffuse reflectance spectroscopy. The absorption edge has been first argued at about 670 nm, *i.e*. 1.85 eV on films of different thicknesses (Figure S2, SI). The band gap has been then better estimated from the extrapolation of the linear part of [*F*(*R*)*h*ν]^2^ where *F*(*R*) is the Kubelka–Munk function *F*(*R*) = (1 – *R*)^2^/2*R*, as reported in Figure 2b. for an oriented Cs_3_Bi_2_I_9_ film. A clear absorption edge is observed corresponding to a direct band gap of about 2.0 eV. Such a value is in agreement with the only paper reporting thin films of Cs_3_Bi_2_I_9_ prepared by spin coating where, however, the authors observed a more structured absorption edge identifying a direct and an indirect band gap.^25^ The indirect gap edge is estimated at 1.96 eV (blue curve) from the indirect gap extrapolation of the linear part of [*F*(*R*)*h*ν]^1/2^.

**Figure 3:**
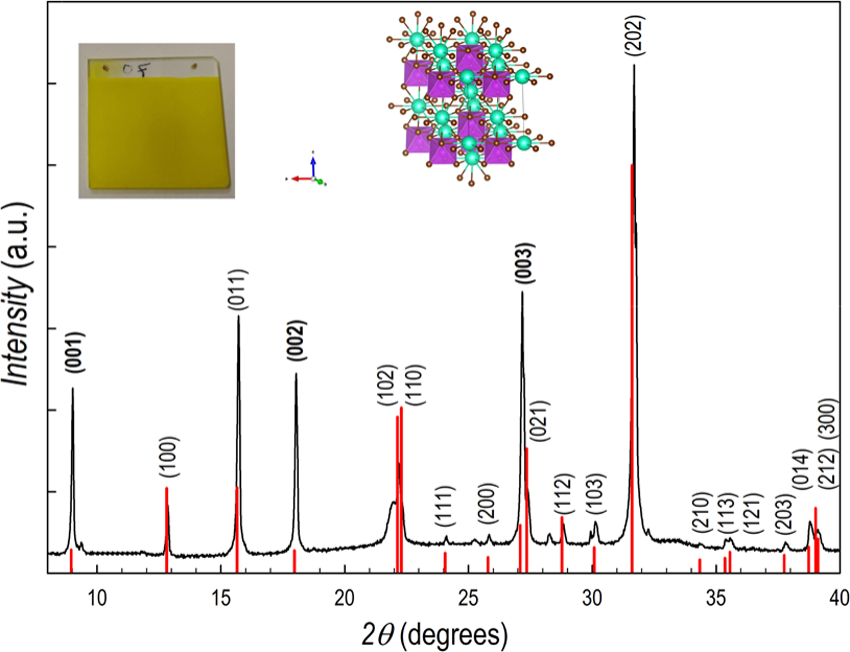
XRD pattern of Cs_3_Bi_2_Br_9_ film superimposed to the calculated pattern of the Cs_3_Bi_2_Br_9_ crystal structure (red lines), inset: from the left: typical aspect of deposited film and sketch of the crystal structure of Cs_3_Bi_2_Br_9_.

Cs_3_Bi_2_Br_9_ films have been deposited starting from stoichiometric amounts of CsBr and BiBr_3_ followed by thermal treatment at 200 °C for 2 h under mild dynamic vacuum (as for the Cs_3_Bi_2_I_9_ material). This approach allowed us to prepare single-phase, highly crystalline, Cs_3_Bi_2_Br_9_ films, as shown in Figure 3, with the typical aspect of the yellow film reported in the inset. Cs_3_Bi_2_Br_9_ films grow in the trigonal crystal structure (*P*3̅*m*—JCPDS card 01-070-0493) as sketched in the inset of the figure, with a slight preferential growth along the (00*l*) direction (Miller indexes in bold in Figure 3) as can be inferred by comparing the experimental pattern with the calculated one (vertical red lines).

**Figure 4:**
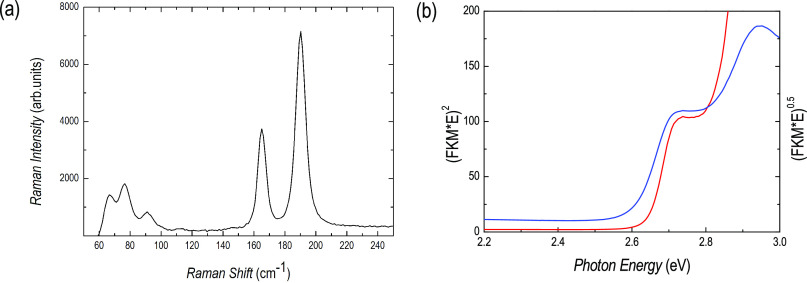
(a) Raman scattering spectrum of the single-phase Cs_3_Bi_2_Br_9_ film and (b) optical absorption edge of the same film (direct gap red curve and indirect gap blue curve extrapolations).

Raman spectroscopy has also been used in this case to further probe the film structure. Figure 4a shows the Raman spectrum of a single-phase Cs_3_Bi_2_Br_9_ film which is in excellent agreement with the published data.^24^ Indeed, the two expected A_1g_ and E_g_ normal modes give strong Raman features centered at ∼190 and 165 cm^–1^, respectively, as a result of Bi–Br vibrations inside the corner-sharing [BiBr_6_]^3–^ octahedra. Additional weaker Raman features are correctly measured at 91, 76, and 66 cm^–1^. The high quality of the obtained structure is confirmed by the full width at half-maximum (FWHM) values of A_1g_ and E_g_ bands (6.9 and 5.3 cm^–1^ respectively), very close to the values obtained for single-crystals.^26^

The optical response is blue-shifted with respect to Cs_3_Bi_2_I_9_, as shown in Figure 4b, and is characterized by a sharp edge corresponding to a band gap of about 2.64 eV to be compared to the value of 2.67 eV reported for the only available Cs_3_Bi_2_Br_9_ film study in the current literature.^2^ Indirect edge is at 2.6 eV (blue curve). The presence of a direct and indirect band gap in Cs_3_Bi_2_Br_9_ has been described in detail by Zhang and co-workers.^27^

So far the first successful application of RF-magnetron sputtering to grow high-quality, single-phase Cs_3_Bi_2_I_9_ and Cs_3_Bi_2_Br_9_ films has been demonstrated, also reporting the first vapor phase growth on these Bi-based perovskite derivatives. The two compositions have distinct crystal structures and optical properties, which, as shown in the next section, can be tuned by halide alloying, also providing a demonstration of the effectiveness of the sputtering method to grow mixed phases.

### Mixed Cs_3_Bi_2_(I_1–*x*_Br*_x_*)_9_ (0 ≤ *x* ≤ 1) Films

Mixed Cs_3_Bi_2_(I_1–*x*_Br_*x*_)_9_ (0 ≤ *x* ≤ 1) have been grown by RF-magnetron sputtering according to the experimental conditions.

**Figure 5:**
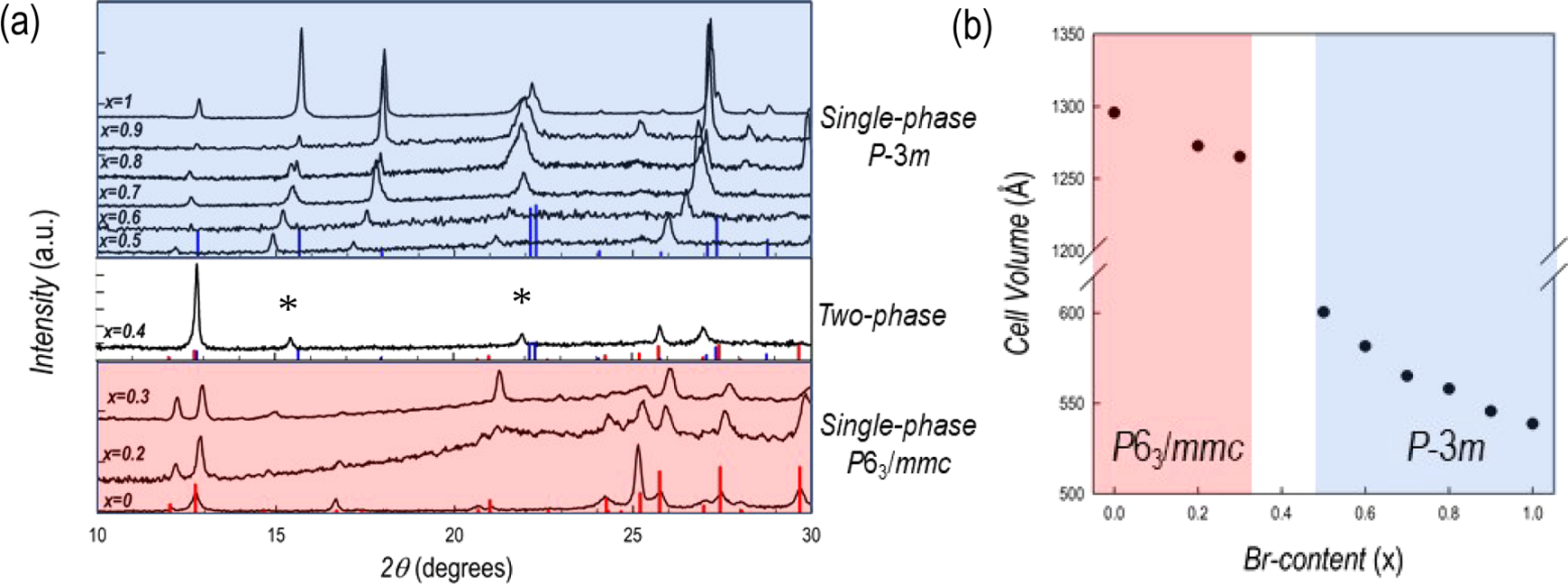
(a) XRD pattern of the Cs_3_Bi_2_(I_1–*x*_Br_*x*_)_9_ (0 ≤ *x* ≤ 1) series of films; (b) trend of lattice volume of Cs_3_Bi_2_(I_1–*x*_Br_*x*_)_9_ (0 ≤ *x* ≤ 1) films as a function of the Br content.

Figure 5a reports the XRD patterns of a series of mixed films with an average thickness of about 300 nm as a function of *x* (bromide content). energy-dispersive X-ray (EDX) analysis was used to determine the effective Br/I content, which was very close to the nominal one with deviations on the order of ±5%. EDS analysis also confirmed the good atomic homogeneity of the prepared films.

For the range 0 ≤ *x* ≤ 0.3 the crystal structure of the mixed compositions is the same as that of Cs_3_Bi_2_I_9_ (*P*6_3_/*mmc*), with the diffraction lines of the samples showing a progressive shift toward higher angle (smaller lattice parameters) on increasing the Br amount. At *x* around 0.4, the XRD pattern shows the presence of the characteristic diffraction peaks of Cs_3_Bi_2_I_9_ (with significant preferential orientation along the 00*l* reflections) together with the appearance of diffraction peaks related to the trigonal crystal structure of Cs_3_Bi_2_Br_9_ (*P*3̅*m*, marked with an asterisk in Figure 5a) which, in addition, are found at significantly lower angles with respect to the reference structure (vertical bluelines of the middle part of Figure 5a), indicating a bigger unit cell due to iodide presence. By further increasing the Br amount, *x*, to 0.5, single-phase samples in mixed system Cs_3_Bi_2_(I_1–*x*_Br_*x*_)_9_ are found. Now, the crystal structure is compatible with that of Cs_3_Bi_2_Br_9_. As it can be observed in the top panel of Figure 5a, and with reference to the reflection of Cs_3_Bi_2_Br_9_ (vertical blue bars), the diffraction peaks of the mixed compositions in the 0.5 ≤ *x* ≤ 0.9 range are shifted to lower angles as a result of the presence of bigger I which increases the cell volume. The trend of lattice volume, determined by profile matching of the diffraction patterns, for the series of films reported in Figure 5a is shown in Figure 5b. A roughly linear trend of *V*, with different slopes, in the two regions of phase stability is observed as a function of the bromide content.

The present results mark a transition from hexagonal to trigonal symmetry at about *x* = 0.3, *i.e*. Cs_3_Bi_2_I_6.3_Br_2.7_, a small intermediate region of mixed-phase samples, and another region of single-phase mixed compositions with trigonal symmetry from Cs_3_Bi_2_I_4.5_Br_4.5_ to Cs_3_Bi_2_Br_9_. The data reported on spin-coated films by Yu et al. evidenced a similar phase transition at a composition equal to that of Cs_3_Bi_2_I_6_Br_3_, indicating the existence of mixed samples around Cs_3_Bi_2_I_7_Br_2_, while alloying in the analogous system in the form of nanocrystals occurs at Cs_3_Bi_2_I_3.6_Br_5.4_ in excellent agreement with the present data.^2^

**Figure 6:**
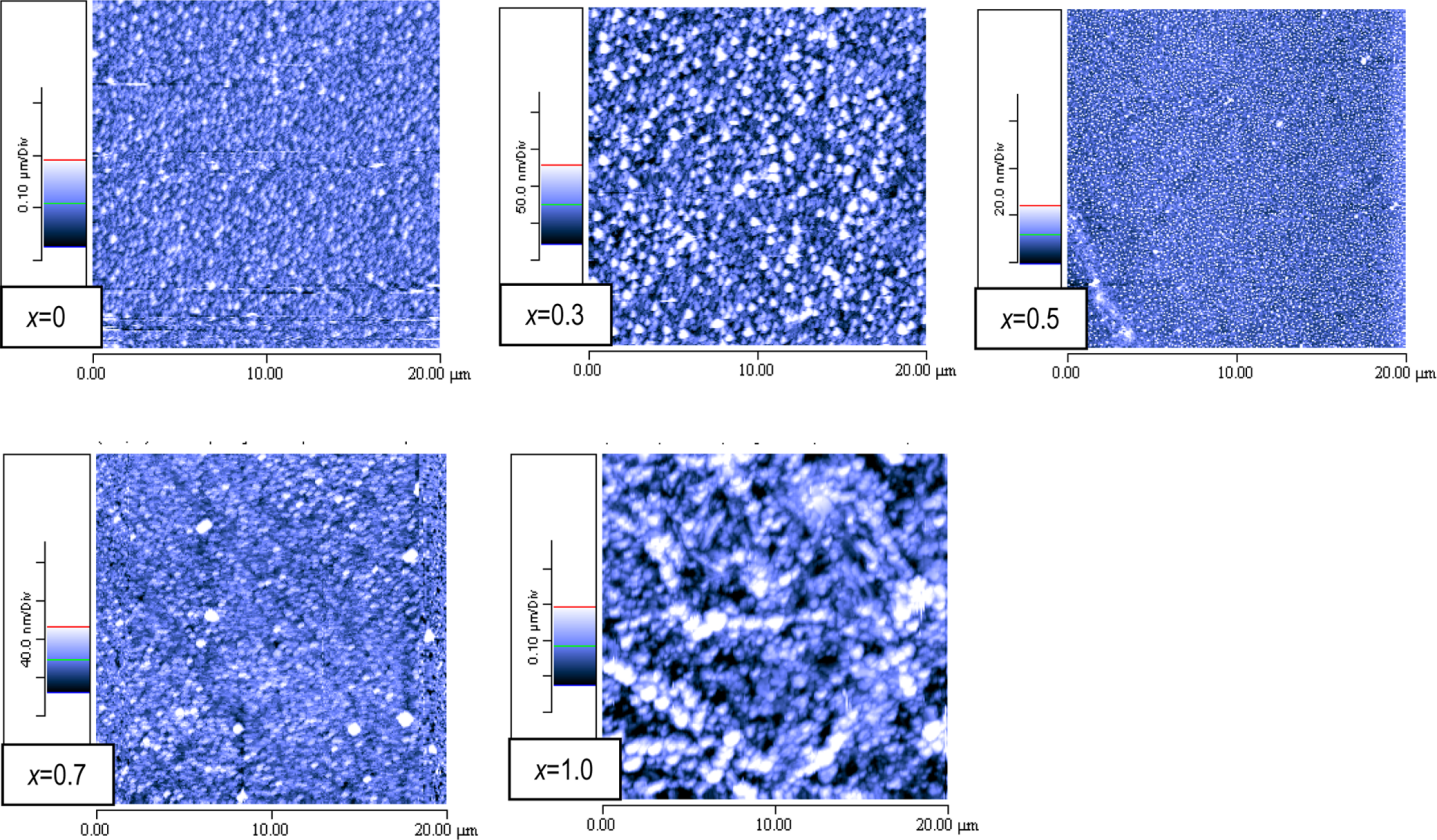
AFM images on 20 × 20 μm^2^ area for Cs_3_Bi_2_(I_1–*x*_Br_*x*_)_9_ samples for *x* = 0, 0.3, 0.5, 0.7, and 1.

Morphological characterization of some selected films of the Cs_3_Bi_2_(I_1–*x*_Br_*x*_)_9_ (0 ≤ *x* ≤ 1) series has been assessed by atomic force microscopy (AFM). In Figure 6 are reported the images collected on 20 × 20 μm^2^ area for films at *x* = 0, 0.3, 0.5, 0.7, and 1. Images of smaller and larger areas are reported in the SI. From the 90 × 90 μm^2^ area (Figure S1) images it is evident that the RF-magnetron sputtering method provides a very good substrate coverage. The root mean square (RMS) roughness spans from 15 to 60 nm, with no clear trend as a function of the Br content with a value of around 30 nm for Cs_3_Bi_2_I_9_ and around 60 nm for Cs_3_Bi_2_Br_9_. From the images in Figure 6 (and the 4 × 4 μm^2^ in the SI) it is possible to note that the films are composed of small particles of about 50–300 nm, depending on the composition (*e.g*., average grain size of around 40–70 nm for Cs_3_Bi_2_I_9_ and around 200–300 nm for Cs_3_Bi_2_Br_9_). In general, there are no significant differences in the film morphology, apart from the slightly less compacted film obtained for Cs_3_Bi_2_Br_9_.

**Figure 7:**
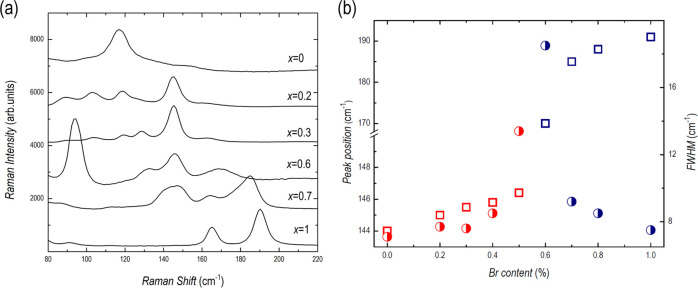
(a) Raman spectra at room temperature of different mixed samples plotted with the two end-members and (b) peak position (open symbols) and linewidth (fully symbols) for the Raman bands corresponding to A′ mode in Cs_3_Bi_2_I_9_ (squares) and to A_1g_ mode in Cs_3_Bi_2_Br_9_ (circles).

The behavior derived from XRD results for mixed samples is further confirmed by Raman data. Room temperature Raman spectra have been registered for all of the investigated samples in the region of 50–1000 cm^–1^. In Figure 7a, for ease of viewing, a selection of mixed samples is reported in the region 80–220 cm^–1^ where Raman yield can be measured. The Raman spectra from the two end-members are also reported for comparison. The spectra in Figure 7a result by averaging all spectra obtained from linear scans as described in the SI. This protocol allowed to check the homogeneity of the Raman behavior of the deposited films from different sample regions. In particular, for *x* < 0.4 and >0.6, the films exhibit a higher degree of homogeneity, as can be seen in Figure S3 where the result from a linear scan of Raman mapping from a sample with *x* = 0.3 is reported as a representative example.

The initial substitution of I by Br leads to a Raman spectrum dominated by the typical A′ mode due to terminal Bi–I symmetric vibrations in Cs_3_Bi_2_I_9_, different from what was already observed for the pure sample, whose spectrum is mainly due to the mode at about 117.0 cm^–1^ attributed to terminal Bi–I asymmetric vibrations. This fact confirms that the addition of low amounts of Br leads to films with a crystal structure equal to that of Cs_3_Bi_2_I_9_ but with different orientations, in accordance with the XRD data. Increasing the Br alloying leads to increasing Raman activity at energies higher than 160 cm^–1^. A very weak signal at around 162 cm^–1^ is already present in the spectrum for the sample with *x* = 0.3 and becomes more and more pronounced with a clear blue-shift with increasing Br content. In the range of 0.3 < *x* < 0.6 it is possible to observe Raman features pertinent to both crystal structures. Indeed, Figure S4 reports the Raman spectrum in the region of 100–200 cm^–1^ obtained for the sample with *x* = 0.5 during a linear scan. The data are interpolated by a superposition of six Lorentzian curves evidencing a mixing between the two phases. It is important to underline that this kind of spectra are in any case rarely observed and by averaging over 15 spectra, the contribution from the minority phase tends to be smeared out. At higher Br amounts, the Raman activity in the region of 80–150 cm^–1^ decreases indicating that the contribution from Bi–I vibrations is gradually quenched, while that from Bi–Br is strengthened. The transition between the two different crystal structures can be further appreciated by monitoring Raman band parameters as reported in Figure 7b.

The Raman modes we used as reference are the already mentioned A′ symmetric mode, involving terminal Bi–I vibrations, for Cs_3_Bi_2_I_9_, and the A_1g_ mode due to the Bi–Br vibrations inside the corner-sharing [BiBr6]^3–^ octahedra in Cs_3_Bi_2_Br_9_, peaked in pure samples at 144.0 and 191.0 cm^–1^, respectively. According to Yu et al., the insertion of Br causes a small blue-shift of the A′ phonon energy with the hardening of the mode of about 2 cm^–1^ for the *x* = 0.4 sample.^2^ On the other hand, the linewidth of this Raman mode increases markedly as the result of the disordering due to the Br insertion in the Bi–I vibrations units. At higher Br amounts the Raman fingerprints of the trigonal crystal structure (*P*3̅*m*) appear at around 160 cm^–1^. The broadened and unresolved feature denotes a higher disorder degree which is gradually quenched leading to the fully ordered phase in the pure sample. In this case, the blue-shift of the A_1g_ mode is greater than that observed for the A′ mode but this is consistent with the contraction of cell volume and the involved anion masses. The higher disorder in the mid-range of substitution and the presence of Raman features ascribable to both crystal systems (see Figure S4) indicate the presence of a mixed-phase.

**Figure 8:**
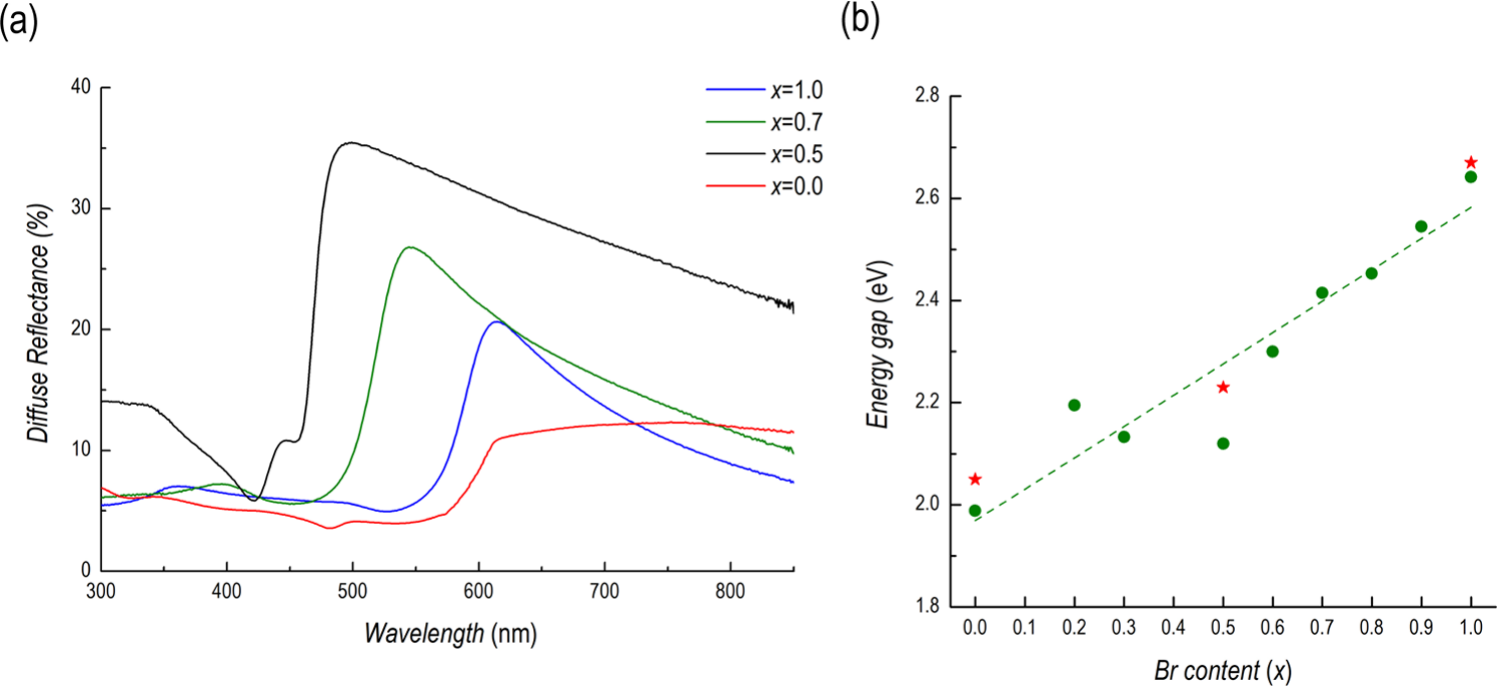
(a) Diffuse reflectance spectra of different mixed Cs_3_Bi_2_(I_1–*x*_Br_*x*_)_9_ samples plotted with the two end-members; (b) fundamental gap energy trend (dots) with linear trend interpolation and a comparison with the literature data (asterisks).

Figure 8a reports the diffuse reflectance spectra of selected films (for ease of representation) for the Cs_3_Bi_2_(I_1–*x*_Br_*x*_)_9_ system showing a clear blue-shift from *x* = 0 to 1. The direct gap energy for all of the films considered shows a progressive shift of the fundamental absorption edge toward higher energies with increasing Br content, with a good linear trend all through the visible range (Figure 8b) and with a slight deviation at *x* = 0.5 which could be possibly correlated to the Raman results indicating, locally, a mixed-phase nature of the sample. The monotonic band gap variation on increasing the Br content, as shown in Figure 8b, well follows the literature trend, as experimentally observed by Gu et al. on only three compositions (asterisks in the Figure).^25^

Finally, we report in the present work some preliminary results aiming at testing the photocatalytic properties of Cs_3_Bi_2_I_9_ and Cs_3_Bi_2_Br_9_ films, which have been assessed by determining their ability in the photodegradation of an organic dye by selecting methylene blue (MB) as a representative model compound of this class. As mentioned in the Introduction section, Cs_3_Bi_2_I_9_ and Cs_3_Bi_2_Br_9_ have attracted significant recent interest due to their relevant photoactivity, which, however, to date has been determined only on particualte samples.^18,19^

**Figure 9:**
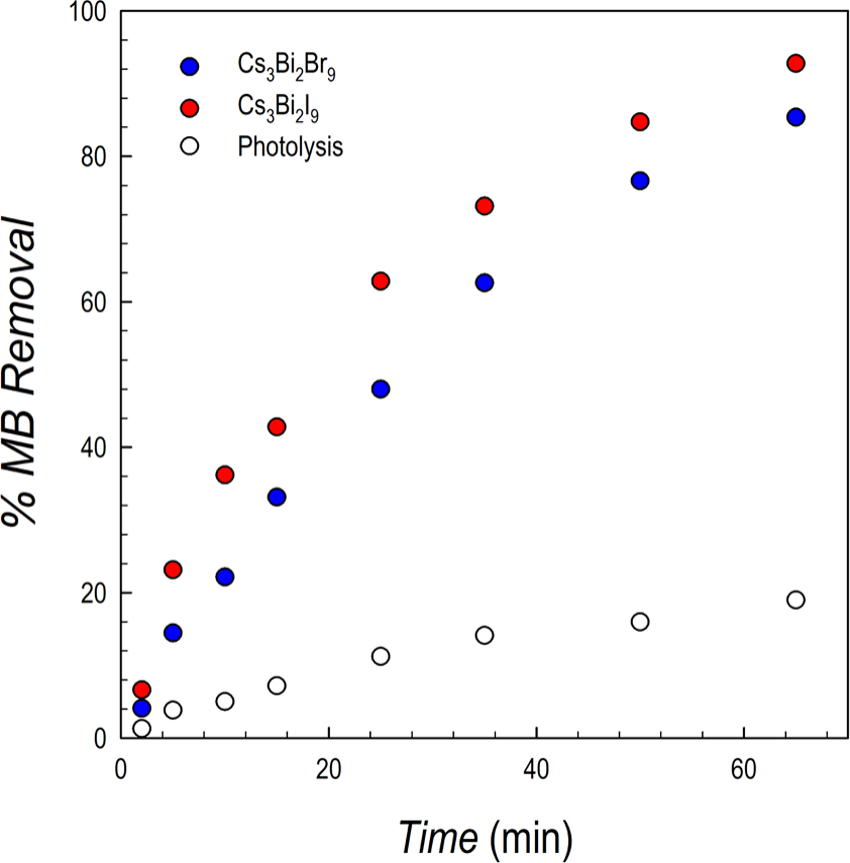
Percent of MB removal as a function of irradiation time for Cs_3_Bi_2_I_9_ (red dots) and Cs_3_Bi_2_Br_9_ (blue dots) films, compared to the photolysis effect (empty dots); conditions: 0.5 g L^–1^ catalyst, 250 W m^–2^ simulated solar light. Relative standard deviation (RSD) < 10% (*n* = 3).

Figure 9 shows the variation of the percentage of MB removal (calculated as *C*_0_ – *C*/*C*_0_ where *C*_0_ is the initial concentration) as a function of irradiation time, compared to that of direct photolysis (see details in the SI).

It is clear from Figure 9 that the two films show relevant activity in the degradation of MB which provides the first evidence of photoactivity by Cs_3_Bi_2_Br_9_ and Cs_3_Bi_2_I_9_ thin films. Slightly different behavior in the MB degradation between the two compositions may be the result of morphology/grain size effects (see AFM data above). The degradation of MB was also investigated concerning the kinetics. For quantitative evaluation, experimental data for MB photodegradation were fitted to different kinetic equations to determine the suitable model to represent the kinetics of degradation.^28^ Plotting the natural logarithm of the ratio between the original concentration and the concentration after photocatalytic degradation ln([MB]_0_/[MB]*_t_*) *versus* the irradiation time (min) provided a linear relationship, as shown in Figure S5. The apparent first-order rate constants (min^–1^) were 0.0295 and 0.0389 for Cs_3_Bi_2_Br_9_ and Cs_3_Bi_2_I_9_ films, respectively. Concerning the mechanism of MB degradation, previous data on bulk Cs_3_Bi_2_Br_9_ and Cs_3_Bi_2_I_9_ indicated that MB degradation occurs *via* the formation of hydroxyl radicals.^29,30^ These promising results open the way to further explore the application of Bi-based perovskites in a series of relevant photocatalytic reactions.

## Conclusions

In the present paper, we provide and demonstrate an efficient vapor deposition route for the preparation of single-phase, high-quality films of Cs_3_Bi_2_Br_9_ and Cs_3_Bi_2_I_9_ based on RF-magnetron sputtering, which is a simple and effective method making use of a single target source. The films have been characterized in terms of their crystal structure, morphology, optical and Raman spectra, and by assessing preliminary photocatalytic activity by investigating the solar-driven MB degradation. RF-sputtering has also been successfully employed to deposit mixed I/Br films Cs_3_Bi_2_(I_1–*x*_Br_*x*_)_9_ (0 ≤ *x* ≤ 1), confirming the suitability of this approach in preparing alloyed compositions with a very good stoichiometry control, as well. A structural phase transition has been observed at about *x* = 0.4, as confirmed by X-ray diffraction, with a scaling of lattice parameters with the halide content. Raman spectroscopy was revealed to be effective in following the structural evolution of mixed compositions. Finally, a variation of the fundamental band gap from 2.0 eV for Cs_3_Bi_2_I_9_ to about 2.64 eV for Cs_3_Bi_2_Br_9_ has been observed with the tuning of *E*_g_ between the two end-members achieved in the mixed compositions. To conclude, this paper demonstrates the efficiency of sputtering in preparing thin films of all-inorganic lead-free Bi-based perovskites which are of huge interest for the wide community of photovoltaics, optoelectronics, and more recently, photocatalysis. As we already demonstrated for other metal halide perovskites, sputtering seems to be a method to be extended to several systems to further boost the scale-up of perovskite-based technology, in particular for all-inorganic systems which face relevant problems in wet-chemistry depositions.^21−23^

## Experimental Section

### Film Deposition

All of the thin films were deposited by means radio frequency (RF) magnetron sputtering on substrates made by cutting microscopy slides (1 mm thick) in 25 × 25 mm^2^ pieces. The substrates were mechanically cleaned with 2-propanol (Aldrich, ≥99.7%), sonicated in the same solvent for 15 min, and heated at 200 °C on a hot plate just before deposition. The targets (diameter 5.08 cm, thickness 1 mm) were made of pressed powders of stoichiometric CsBr/CsI/BiI_3_/BiBr_3_ mixtures (Aldrich, 99.9%). Overall, the starting mass of the target was about 10 g.

Deposition parameters were: (i) target-to-substrate distance, 10 cm, (ii) RF-power, 50 W, (iii) argon pressure, 2.3 × 10^–2^ mbar, and (iv) argon flux 20 sccm. The depositions were carried out in power-control mode. Film thickness was determined using a P-6 stylus profilometer KLA Tencor equipped with a silicon tip (tip radius 2 μm; applied force 2 mg). After the deposition, the films have been heated and cooled in a vacuum using a BÜCHI glass drying oven for 2 h at 200 °C.

### XRD Diffraction

The structural properties of the deposited thin films were characterized by X-ray diffraction (XRD) using a Bruker D8 Advance instrument (Cu radiation) in a Bragg–Brentano setup.

### EDX and Scanning Electron Microscopy (SEM)

EDX analysis and microstructural characterization of the samples were performed using a high-resolution scanning electron microscope (SEM, TESCAN Mira 3) operated at 25 kV.

### AFM

Atomic force microscopy (AFM) images were obtained with an AutoProbe CP microscope (ThermoMicroscopes-Veeco), operating in tapping mode, by means of sharpened silicon tip Nanosensors (resonance frequency: 300 kHz; force constant: 40 N m^–1^). For each analyzed film, scans from 90 μm × 90 μm to 4 μm × 4 μm have been carried out with the scan rate ranging from 0.5 to 1 Hz. A standard second-order flatten processing of the images has been performed to correct the scanner nonlinearity.

### Absorption and Diffuse Reflectance Spectroscopy

Ultraviolet–visible–near infrared (UV–vis–NIR) optical measurements were performed under ambient conditions using a Varian Cary 6000i spectrophotometer equipped with a double monochromator, a deuterium lamp and a tungsten filament lamp as light sources, and a photomultiplier (UV–vis) and an InGaAs photodiode (NIR) as detectors. The spectral range was 200–1800 nm, in a step of 1 nm. Both near-normal absorption spectra and diffuse reflectance spectra with a 110 mm diameter integrating sphere were recorded.

### Raman Spectroscopy

Micro-Raman measurements were carried out at room temperature using a Labram Dilor spectrometer equipped with an Olympus microscope HS BX40. The 632.8 nm light from a He–Ne laser was employed as excitation radiation. The samples, mounted on a motorized *xy* stage, were tested with a 50× objective and with a laser spot of ∼1.5 μm diameter. The spectral resolution was about 1 cm^–1^. A cooled charge-coupled device (CCD) camera was used as a detector and the typical integration times were about 2 min. The sample phase homogeneity was verified by performing linear scanning over a length of about 30 μm in three different sample regions. From these scans, an average spectrum for each sample has been derived. These spectra were processed by best-fitting procedures based on Lorentzian functions.

### Methylene Blue Photodegradation

Methylene blue degradation was conducted in a batch setup. Methylene blue solution (3 mL, 0.5 g L^–1^) was placed in a quartz cuvette together with the catalyst film, sputtered onto a 0.8 cm × 4.0 cm glass substrate. The progress of the reaction was monitored measuring the absorption of the solution at 664 nm. The catalyst film was placed in a direction perpendicular to the irradiation path. Irradiation was conducted in a solar box equipped with a Xe lamp and the irradiance was set to 250 W m^–2^, to avoid excessive degradation of the methylene blue during photolysis (without the catalyst). Before the start of the irradiation, the filled cuvette was kept in the dark for half an hour to enable the establishment of an adsorption–desorption equilibrium between methylene blue and the thin film. The experiments were repeated three times (RSD < 10%).

## Supporting Information Available

Additional AFM and Raman and diffuse reflectance spectra (PDF)
