# Supplementary material for: Optical and Structural Property Tuning in Physical Vapor Deposited Bismuth Halides Cs3Bi2(I1–xBrx)9 (0 ≤ x ≤ 1)
Source: Inorg Chem. 2021 Sep 2;60(18):14142–50. doi: 10.1021/acs.inorgchem.1c01545 (PMC8456412; doi:10.1021/acs.inorgchem.1c01545)
Supplement: Supplementary file 1 — ic1c01545_si_001.pdf [file ic1c01545_si_001.pdf]

SUPPORTING INFORMATION

Optical and Structural Properties Tuning in Physical Vapor Deposited Bismuth Halides $Cs_3Bi_2(I_{1-x}Br_x)_9$ ($0 \leq x \leq 1$)

*Sara Bonomi,[a] Pietro Galinetto,[b] Maddalena Patrini,[b] Lidia Romani,[a] Lorenzo Malavasi[a,]**

[a]Department of Chemistry and INSTM, University of Pavia,Via Taramelli 16, Pavia, 27100, Italy

[b]Department of Physics, University of Pavia, Via Bassi 6, Pavia, 27100, Italy

Corresponding Author

Lorenzo Malavasi, email: lorenzo.malavasi@unipv.it; tel. +39 382 987921

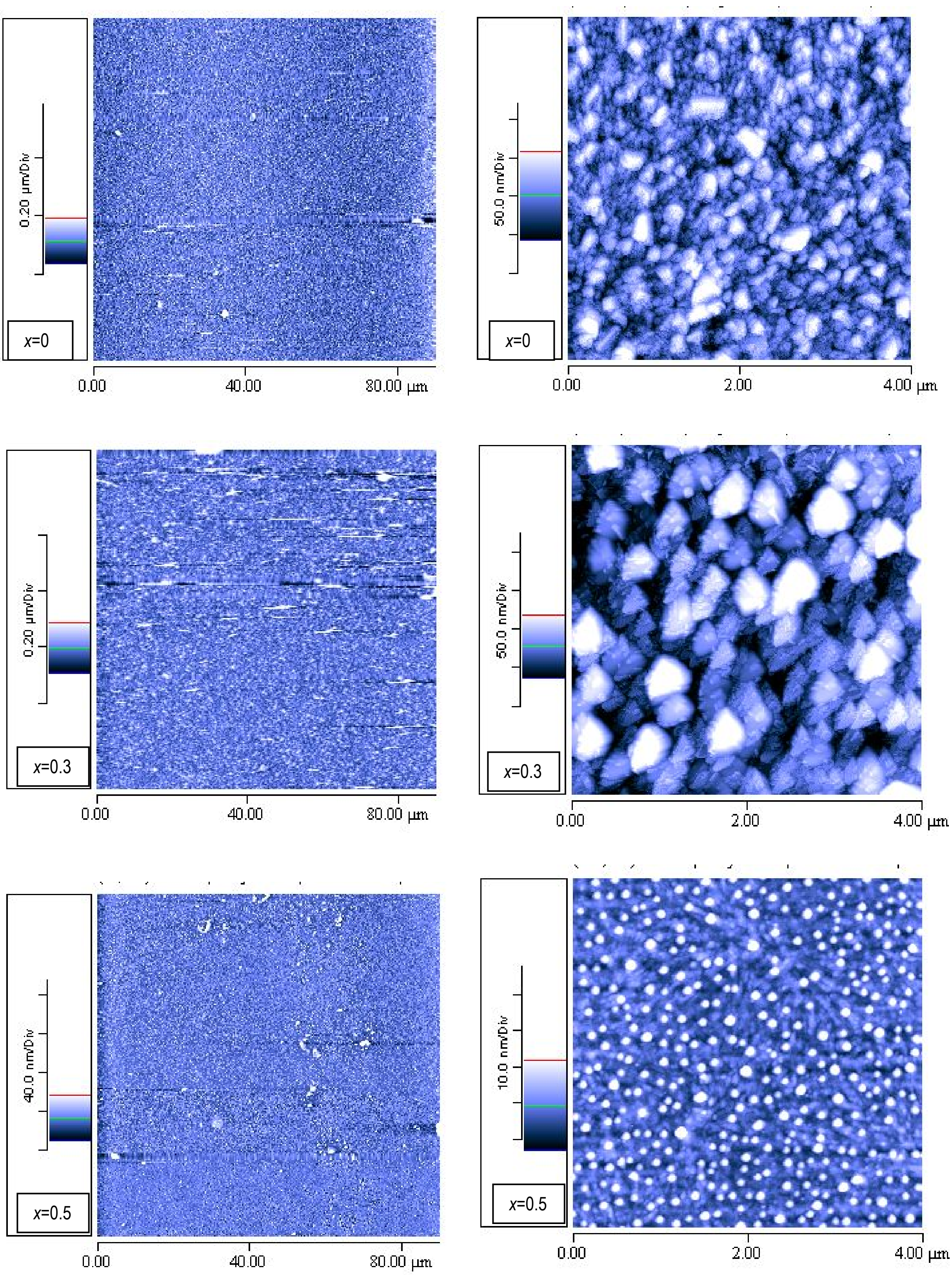
0.20 µm/Div
x=0
0.00
40.00
80.00 µm
50.0 nm/Div
x=0
0.00
2.00
4.00 µm
0.20 µm/Div
x=0.3
0.00
40.00
80.00 µm
50.0 nm/Div
x=0.3
0.00
2.00
4.00 µm
40.0 nm/Div
x=0.5
0.00
40.00
80.00 µm
10.0 nm/Div
x=0.5
0.00
2.00
4.00 µm

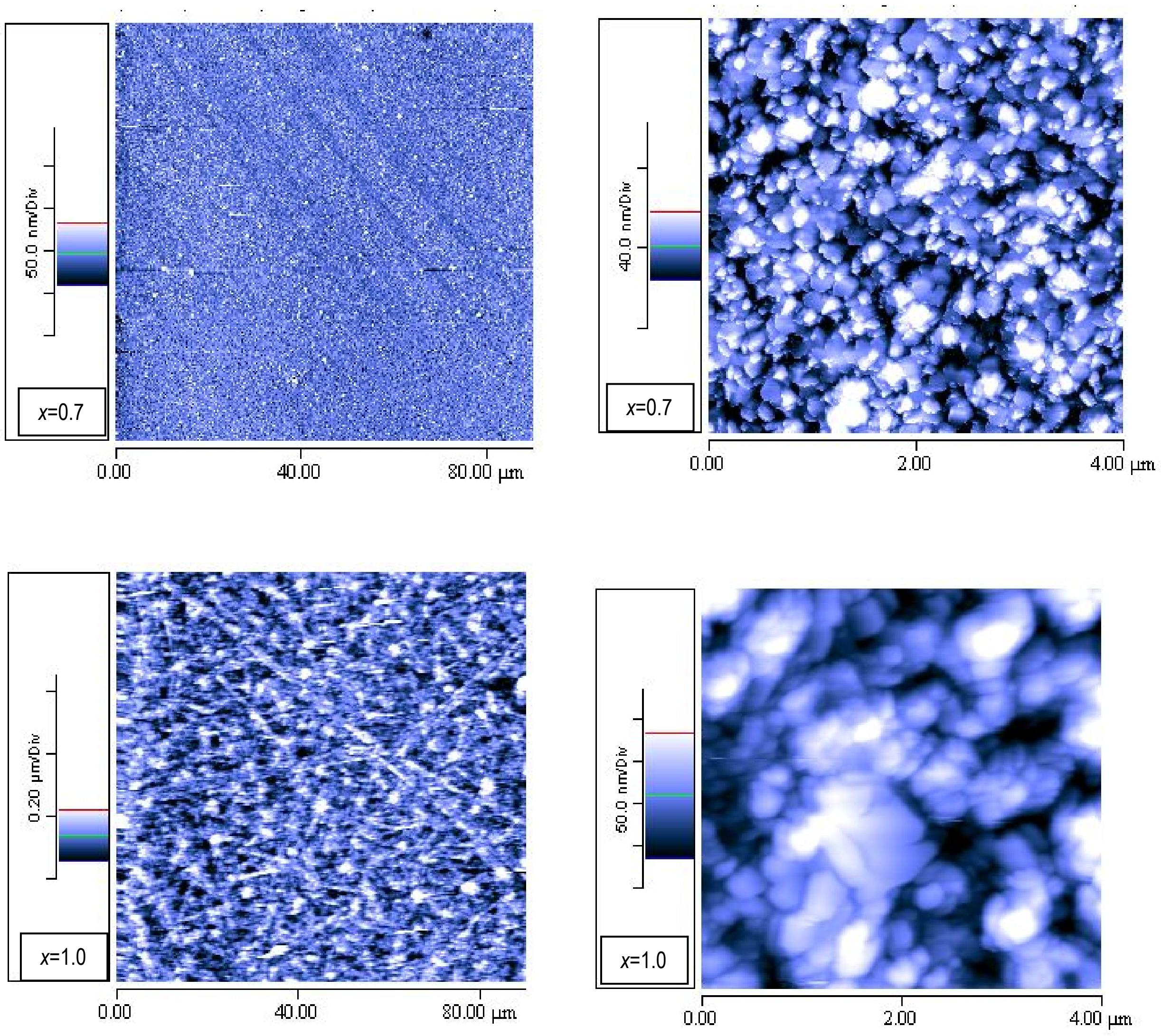

Figure S1. AFM images on 90 × 90 µm and 4 × 4 µm area for $Cs_3Bi_2(I_{1-x}Br_x)_9$ samples for x=0, 0.3, 0.5, 0.7 and 1.

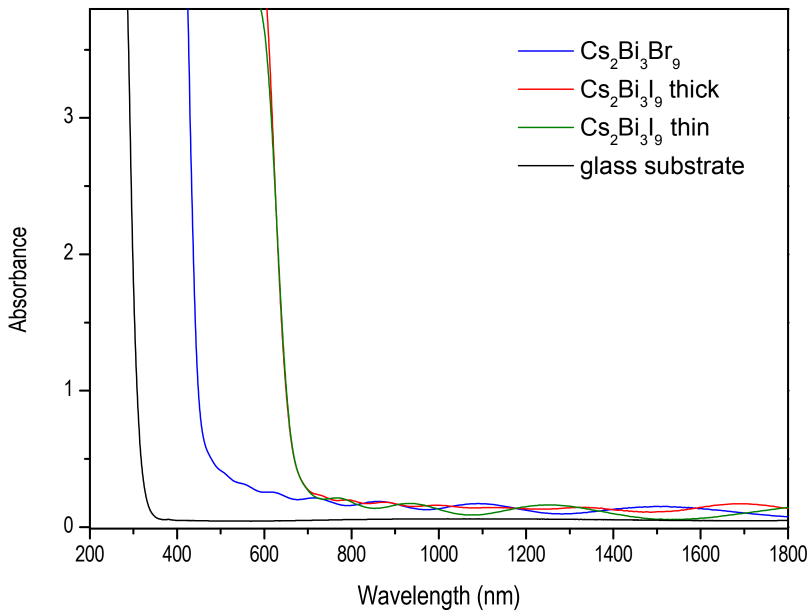

Figure S2. Absorbance spectra of non-oriented end-member films of $Cs_2Bi_3Br_9$ 0.8 μm thick and $Cs_2Bi_3I_9$ (two different thicknesses of about 0.7 μm and 1.25 μm) as compared to the glass substrate response. Clear direct absorption edges are derived from Tauc plot at about 470 nm and 670 nm, respectively.

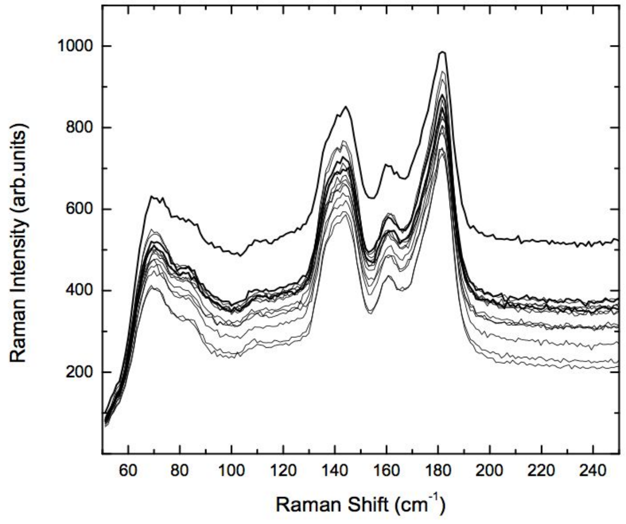

Figure S3. Raman spectra obtained from a linear scan (see text) from the sample with x=0.3.

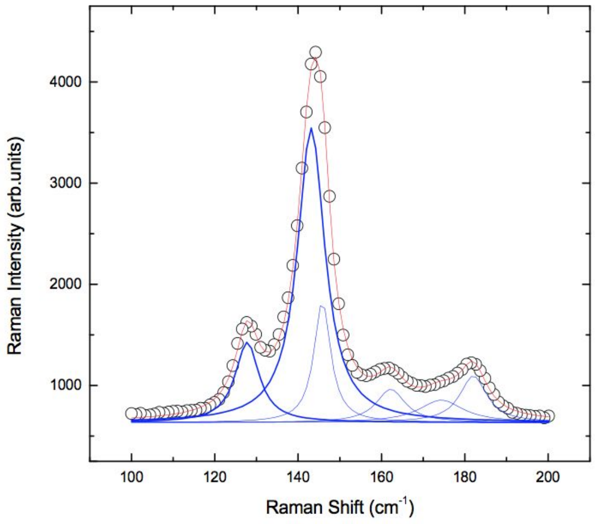

Figure S4. Raman spectrum in the region 100-200 cm^{-1} for the sample x=0.5. The red line is the result of best-fitting procedure performed using six lorentzian curves. The peak positions are consistent with the expected energies for the two crystal structures.

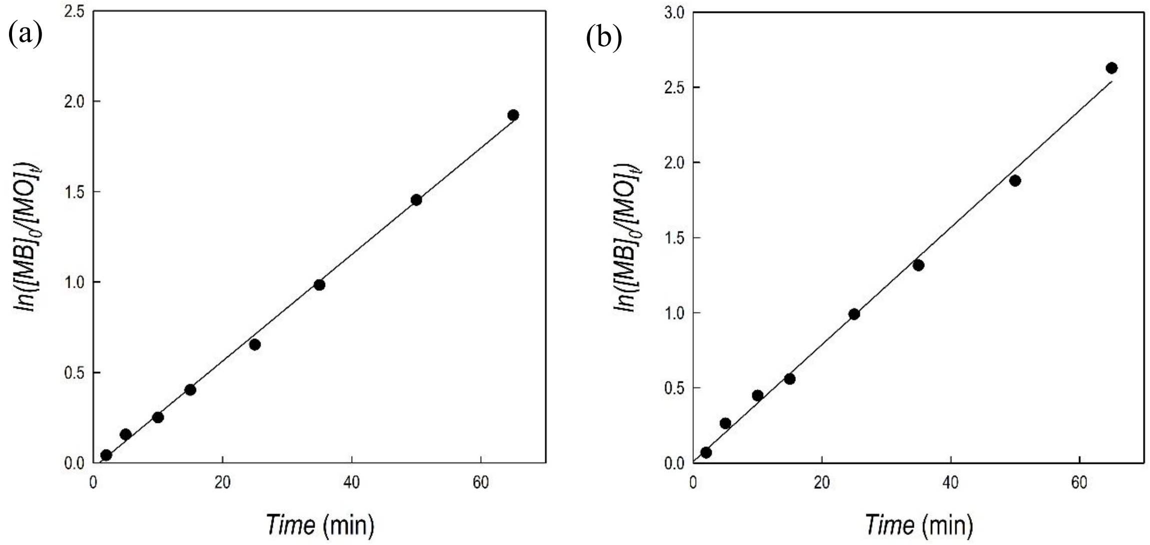

Figure S5. Plot of the fit of ln($[MB_0/[MB]_t$) versus the irradiation time (min) for (a) $Cs_2Bi_3Br_9$ and (b) $Cs_2Bi_3I_9$ films.
